# Endothelial Activation Phenotypes and Interleukin-6 Response After Therapeutic Plasma Exchange in Severe COVID-19-Associated Sepsis: A Retrospective Cohort Study

**DOI:** 10.3390/diseases14040123

**Published:** 2026-03-28

**Authors:** Nicoleta Sgavardea, Ovidiu Bedreag, Greeshmasree Kambam, Tamara Mirela Porosnicu, Ciprian Gîndac, Claudiu Barsac, Cristian Oancea, Patricia Hogea, Alexandru Crisan, Voichita Elena Lazureanu

**Affiliations:** 1 Doctoral School, “Victor Babes” University of Medicine and Pharmacy, Eftimie Murgu Square 2, 300041 Timisoara, Romania; nicoleta.cotaia@umft.ro; 2 Anaesthesia and Intensive Care Research Center, Faculty of Medicine, “Victor Babes” University of Medicine and Pharmacy, Eftimie Murgu Square 2, 300041 Timisoara, Romania; bedreag.ovidiu@umft.ro (O.B.); claudiu.barsac@umft.ro (C.B.); 3 Frank H. Netter School of Medicine, Quinnipiac University, Hamden, CT 06473, USA; greeshmasree.kambam@hhchealth.org; 4 Center for Research and Innovation in Precision Medicine of Respiratory Diseases (CRIPMRD), “Victor Babes” University of Medicine and Pharmacy, Eftimie Murgu Square 2, 300041 Timisoara, Romania; oancea@umft.ro (C.O.); hogea.patricia@umft.ro (P.H.); 5 Pulmonary Rehabilitation Center, Clinical Hospital of Infectious Diseases and Pulmonology, “Victor Babes”, Gheorghe Adam Street 13, 300310 Timisoara, Romania; crisan@umft.ro; 6 Research Center for Assessment of Human Motion, Functionality, and Disability, “Victor Babes” University of Medicine and Pharmacy, Eftimie Murgu Square 2, 300041 Timisoara, Romania; 7 Discipline of Infectious Disease, “Victor Babes” University of Medicine and Pharmacy, Eftimie Murgu Square 2, 300041 Timisoara, Romania; lazureanu.voichita@umft.ro

**Keywords:** COVID-19, sepsis, plasma exchange, interleukin-6, fibrinogen

## Abstract

Background and Objectives: Severe COVID-19 frequently fulfills Sepsis-3 criteria and is characterized by thrombo-inflammation and endothelial injury. We evaluated whether a bedside endothelial activation index (EAI = D-dimer/fibrinogen) identifies biologically distinct phenotypes and relates to interleukin-6 (IL-6) response after therapeutic plasma exchange (TPE), and whether baseline IL-6 predicts a ≥50% IL-6 reduction. Methods: Retrospective single-center ICU cohort of adults with SARS-CoV-2 infection, sepsis-related organ dysfunction, and ≥1 TPE session (n = 51). Patients were stratified by median EAI (low vs. high). Outcomes included peri-procedural biomarker/physiology changes (post–baseline), IL-6 responder status (≥50% reduction), correlations with IL-6 reduction (%), and multivariable predictors of response. Results: Compared with low EAI (n = 25), high EAI (n = 26) had higher baseline D-dimer (6.2 vs. 2.2 µg/mL) and lower fibrinogen (2.9 vs. 7.1 g/L) (both *p* < 0.001). Low EAI showed larger CRP decreases (ΔCRP −84.0 vs. −2.3 mg/L; *p* = 0.001) and larger fibrinogen falls (Δ −3.1 vs. −0.4 g/L; *p* < 0.001), while high EAI had larger D-dimer decreases (Δ −2.5 vs. −0.6 µg/mL; *p* = 0.004) and a modest SOFA improvement (Δ −0.3 vs. +0.1; *p* = 0.026). IL-6 responders (n = 20) had higher baseline IL-6 than non-responders (365.2 vs. 47.1 pg/mL; *p* < 0.001). Baseline IL-6 independently predicted response (per doubling: OR 1.94, 95% CI 1.27–2.95; *p* = 0.002), while age reduced odds (OR 0.91/year, 95% CI 0.84–0.99; *p* = 0.032). IL-6 reduction correlated with ΔCRP (ρ = −0.41; *p* = 0.003) and ΔPaO_2_/FiO_2_ (ρ = 0.37; *p* = 0.01). Conclusions: EAI stratifies distinct thrombo-inflammatory patterns around TPE, while baseline IL-6 is the dominant predictor of achieving large IL-6 reductions. To emphasize the novelty and clarify the study objective, this exploratory analysis used a phenotype-stratified framework to test whether a simple bedside endothelial activation index could enrich biological response assessment to adjunctive TPE. The prespecified primary outcome was achievement of a ≥50% IL-6 reduction after completion of the TPE course; secondary outcomes included peri-procedural biomarker, oxygenation, SOFA, and ICU endpoints.

## 1. Introduction

Severe COVID-19 frequently evolves into a syndrome that meets Sepsis-3 criteria—i.e., life-threatening organ dysfunction driven by a dysregulated host response—with a characteristic blend of microvascular injury, endothelial activation, and multi-organ impairment measured pragmatically by trajectories in SOFA components (respiratory, cardiovascular, renal, hepatic, neurologic, hematologic) [1]. In COVID-19, this biology is often framed as “immunothrombosis,” where inflammatory signaling and coagulation amplify one another through endothelial perturbation, platelet–leukocyte crosstalk, and fibrin deposition, producing heterogeneous patterns of micro and macrovascular thrombosis [2].

This thrombo-inflammatory convergence is reflected in routine laboratory signatures: rising D-dimer, variable fibrinogen (often elevated early as an acute-phase reactant, but potentially falling with consumption in advanced disease), and other markers of coagulation imbalance that track clinical deterioration [3,4]. Early clinical cohorts demonstrated that abnormal coagulation parameters were associated with worse prognosis in COVID-19, supporting the concept that the coagulation system is not merely a bystander but a coupled effector arm of severe disease biology [3]. Meta-analytic data further emphasize the magnitude of this signal: elevated D-dimer is associated with substantially higher risk of severe disease and mortality (fourfold higher mortality risk in pooled analyses, depending on study definitions and cutoffs) [5].

Among circulating cytokines, interleukin-6 (IL-6) remains one of the most reproducible correlates of severe COVID-19, consistent with its role in hepatic acute-phase responses, vascular permeability, and downstream inflammatory amplification [6]. Importantly, IL-6 is not uniformly “high” in all critically ill patients—both clinical observation and quantitative syntheses show wide dispersion in IL-6 concentrations across severity strata, implying that single thresholds may be too blunt for selecting adjunctive therapies [6]. The biologic plausibility of targeting this axis is reinforced by randomized platform trials of IL-6 receptor antagonism in critical illness, which demonstrated improved clinical outcomes in appropriately selected, systemically inflamed patients [7,8].

Therapeutic plasma exchange (TPE) has been proposed as a mechanistically “broad-spectrum” adjunct, capable of removing circulating inflammatory mediators and modulating plasma composition (including prothrombotic and endothelial-active proteins) through replacement with donor plasma/albumin [9]. Early interventional cohorts in severe COVID-19 suggested feasibility and biomarker shifts after TPE, but were limited by confounding, timing heterogeneity, and variable co-interventions that complicate inference [9]. More recent controlled evaluations have sharpened the question: randomized trials have shown that plasma exchange can reduce select prothrombotic markers (e.g., FVIII, VWF-related indices) yet may not translate into measurable improvements in respiratory failure progression, thrombotic events, or short-term mortality—highlighting a potential “biomarker–outcome disconnect” when patient biology is not aligned with the therapy’s dominant mechanism [10,11].

A recent critical care review has further highlighted current progress in TPE for critical illness, emphasizing its biologically broad but still selectively applicable role, and underscoring ongoing uncertainties regarding timing, exchange prescription, replacement fluid, and patient selection in sepsis-like hyperinflammatory states [9].

A recurring challenge, therefore, is not simply whether TPE can change laboratory values, but whether it changes them coherently within a biologically meaningful subtype—and whether those changes map onto early organ failure trajectories. In non-COVID sepsis populations, secondary analyses of randomized/cluster-randomized data have also raised caution that TPE may fail to improve organ failure or mortality and may prolong ICU stay, underscoring that extracorporeal “de-bulking” strategies likely require more precise selection frameworks [12]. In parallel, ICU COVID-19 research has increasingly supported the existence of distinct immuno-inflammatory phenotypes (e.g., differential T-cell activation/exhaustion patterns and variable inflammatory set-points) that plausibly influence both baseline biomarker levels and treatment responsiveness [13].

While unsupervised clustering can identify such subgroups retrospectively, bedside application often demands simpler, interpretable indices. D-dimer reflects fibrin formation and breakdown, whereas fibrinogen captures acute-phase substrate availability and coagulation factor reserve—making their ratio an intuitively coupled signal of thrombotic activity relative to substrate abundance. Prior work has shown that the D-dimer/fibrinogen ratio carries prognostic information in hospitalized COVID-19 populations, supporting its use as a compact “thrombo-inflammatory balance” marker [14]. In this learning-focused study, we extend this rationale by operationalizing an “endothelial activation index” (EAI) from the D-dimer/fibrinogen ratio, then stratifying patients by EAI phenotype to examine peri-procedural coagulation dynamics, IL-6 change after TPE, and short-term organ failure signals (SOFA). We further test whether baseline IL-6 remains the dominant predictor of IL-6 response after accounting for EAI and baseline severity, in a disease where endothelial dysfunction is central to multi-organ injury biology [15].

## 2. Materials and Methods

### 2.1. Study Design and Setting

This was a retrospective observational cohort study performed in a single university-affiliated intensive care program where TPE was available as an adjunctive rescue therapy during severe COVID-19–associated sepsis at the institutions affiliated with Victor Babes University of Medicine and Pharmacy, Timisoara. The cohort included critically ill adults who underwent TPE in routine care. For this study, a total of 51 patients were included in the final analysis. The present report adheres to the STROBE (Strengthening the Reporting of Observational Studies in Epidemiology) statement for cohort studies [16].

The study received approval code number 48 and 10 January 2023 approval by The Local Commission of Ethics from the “Pius Brinzeu” Clinical Emergency Hospital from Timisoara, Romania. The Local Commission of Ethics from the “Pius Brinzeu” Clinical Emergency Hospital from Timisoara, Romania operates under article 167 provisions of Law no. 95/2006, art. 28, chapter VIII of order 904/2006; with EU GCP Directives 2005/28/EC, International Conference of Harmonisation of Technical Requirements for Registration of Pharmaceuticals for Human Use (ICH); and with the Declaration of Helsinki—Recommendations Guiding Medical Doctors in Biomedical Research Involving Human Subjects.

Eligible patients were adults (≥18 years) with laboratory-confirmed SARS-CoV-2 infection, sepsis-related organ dysfunction (captured by SOFA), and receipt of ≥1 TPE session. Patients were managed according to local ICU standards, including ventilatory support, hemodynamic resuscitation, and anti-inflammatory/antimicrobial strategies as clinically indicated. Patients with missing key paired measurements (pre-TPE or post-TPE IL-6, D-dimer, fibrinogen, or SOFA) were not included in the analytic dataset.

TPE was delivered using standard apheresis platforms with exchange volumes consistent with routine ICU practice (approximately one plasma volume per procedure, adapted to body size and hemodynamic tolerance). Replacement fluid selection (albumin with or without plasma components) followed the clinician’s judgment and coagulation status. Because the goal was to phenotype biological response rather than compare technical protocols, the primary analytic contrast was baseline phenotype rather than treatment “dose”.

The number of TPE sessions was individualized according to clinical response, coagulation profile, and treating-team judgment, which reflects real-world rescue practice but also introduces protocol heterogeneity.

### 2.2. Data Collection and Definitions

Demographic data included age, sex, and anthropometrics (weight, height, calculated BMI). Comorbidities included diabetes mellitus, arterial hypertension, obesity, and chronic obstructive pulmonary disease. Baseline severity was captured with APACHE II and SOFA. Physiological variables included mean arterial pressure (MAP), PaO_2_/FiO_2_ ratio, lactate, heart rate, and temperature when available at paired timepoints.

Biomarkers were assessed at two clinically aligned timepoints: immediately pre-TPE (baseline) and after completion of the TPE course planned by clinicians (post-TPE). Primary biomarkers included IL-6, CRP, D-dimer, and fibrinogen; supportive markers included ferritin, LDH, leukocytes, lymphocyte metrics, and procalcitonin where available. Changes were defined as Δ = post − baseline. The key phenotype variable was the endothelial activation index (EAI) = D-dimer/fibrinogen at baseline; patients were split into high vs. low EAI using the cohort median. IL-6 response was defined a priori as ≥50% IL-6 reduction (percent change).

All laboratory measurements were obtained through the hospital’s central laboratory as part of routine clinical care using standardized institutional assays. EAI was selected as a composite bedside marker because D-dimer and fibrinogen capture complementary dimensions of thrombo-inflammatory biology—ongoing fibrin turnover and available coagulation/acute-phase substrate—and their ratio was intended to reflect relative endothelial-thrombotic imbalance rather than either analyte in isolation. The primary outcome was achievement of a ≥50% IL-6 reduction after TPE, chosen a priori as a pragmatic marker of substantial biological response in the absence of validated minimal clinically important differences for peri-TPE IL-6 change in severe COVID-19. Secondary outcomes were changes in CRP, D-dimer, fibrinogen, PaO_2_/FiO_2_, SOFA, ICU mortality, ventilator-free days, ICU length of stay, and ventilation duration.

### 2.3. Statistical Analysis

Continuous variables are presented as median (IQR) and categorical variables as n/N (%), with one-decimal formatting. Between-group comparisons (high vs. low EAI; IL-6 responder vs. non-responder) used Mann–Whitney U tests for continuous variables and Fisher’s exact tests for categorical variables. For four-group outcome summaries (phenotype × responder status), continuous outcomes were compared with Kruskal–Wallis tests, and categorical outcomes with χ^2^ tests.

Associations between IL-6 reduction (%) and concurrent changes in clinical/biomarker variables were tested using Spearman’s rank correlation (ρ). Multivariable predictors of IL-6 response were evaluated using logistic regression, reporting adjusted odds ratios (OR), 95% confidence intervals (CI), and *p*-values. Two-sided *p*-values < 0.05 were interpreted as statistically significant.

Because this was a retrospective exploratory cohort, no formal a priori sample size calculation was performed; the study sample comprised all consecutive eligible TPE-treated patients with complete paired data available during the study period. Analyses used a complete-case approach. Patients with missing paired pre-/post-TPE measurements required for the primary endpoint were excluded from the analytic dataset, and no imputation was performed.

## 3. Results

Baseline characteristics are summarized in Table 1. Apart from the expected separation in D-dimer, fibrinogen, and CRP, the two EAI phenotypes were broadly comparable with respect to age, body mass index, timing to first TPE, baseline severity scores, and oxygenation.

Peri-procedural changes are summarized in Table 2. In brief, the low-EAI phenotype showed a more pronounced acute-phase response reduction, whereas the high-EAI phenotype showed a greater D-dimer decrease together with a numerically modest improvement in SOFA. Because the absolute SOFA difference was small, this finding should be interpreted as an early physiologic signal rather than evidence of a large clinical effect.

Baseline predictors and outcomes according to IL-6 responder status are presented in Table 3. Baseline IL-6 was the only variable that clearly differentiated responders from non-responders, whereas exploratory ICU outcomes were not statistically different between groups.

In adjusted analysis, baseline IL-6 remained the dominant predictor of IL-6 response, while older age was associated with lower odds of achieving the prespecified response threshold (Table 4).

IL-6 reduction correlated with concurrent CRP decline and improved oxygenation, whereas correlations with D-dimer, SOFA, MAP, and lactate were weak and not statistically significant (Table 5).

Across the combined phenotype-response strata, the principal between-group difference was the magnitude of IL-6 reduction, whereas the exploratory clinical outcomes remained similar across groups (Table 6).

**Figure 1:**
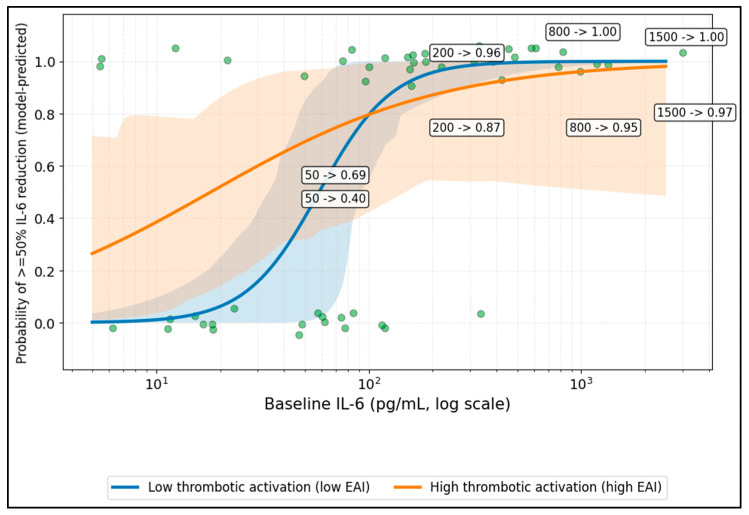
Baseline IL-6 and probability of ≥50% IL-6 reduction, stratified by endothelial activation phenotype (EAI). Shaded areas indicate model uncertainty bands.

Model-based curves suggested phenotype-dependent response probabilities primarily in the low-to-moderate baseline IL-6 range, with progressive convergence at higher IL-6 values (Figure 1). The apparent intersection of the curves should be considered exploratory and not interpreted as a validated treatment-selection cutoff.

**Figure 2:**
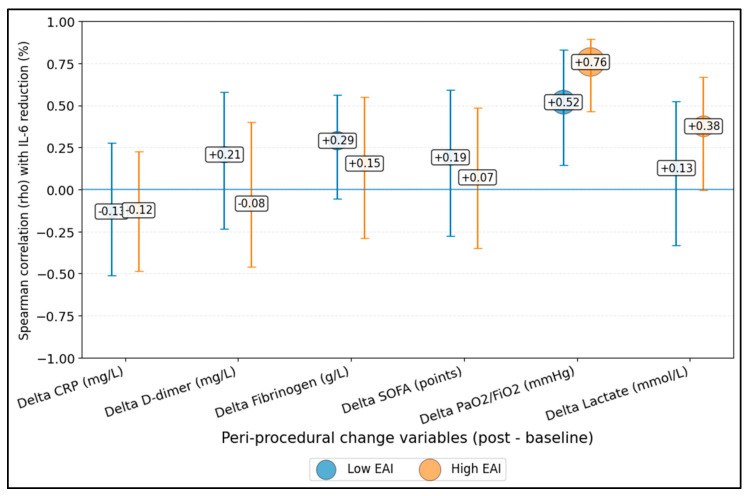
Phenotype-stratified correlations between IL-6 reduction (%) and peri-procedural physiologic/biomarker changes. Δ, post–baseline change; CRP, C-reactive protein; PaO_2_/FiO_2_, arterial oxygen partial pressure to fraction of inspired oxygen ratio.

Figure 2 shows that improvement in oxygenation had the most consistent positive association with IL-6 reduction across both phenotypes, whereas relationships with other biomarkers were weaker and more variable.

**Figure 3:**
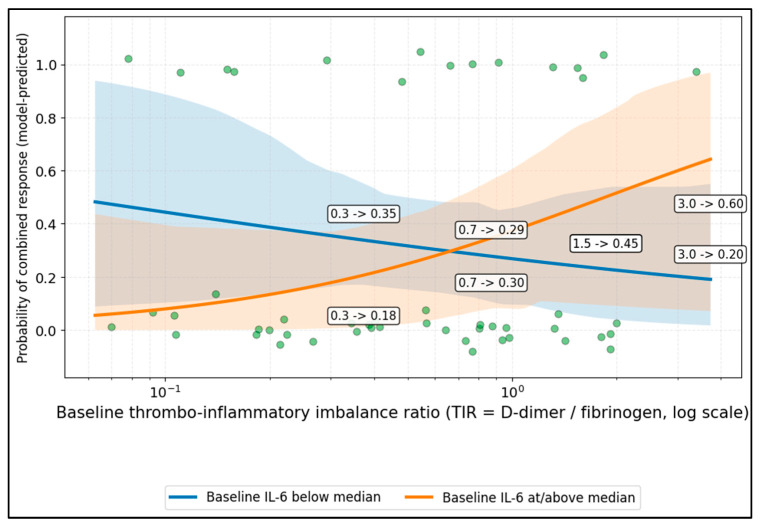
Probability of combined response across endothelial activation index (EAI = D-dimer/fibrinogen), stratified by baseline IL-6. For consistency, this ratio is referred to throughout the revised manuscript as EAI; TIR, thrombo-inflammatory imbalance ratio.

Figure 3 illustrates a crossover interaction between baseline inflammatory burden and EAI in relation to the combined response probability, suggesting that the effect of thrombo-inflammatory imbalance may depend on the starting IL-6 state.

**Figure 4:**
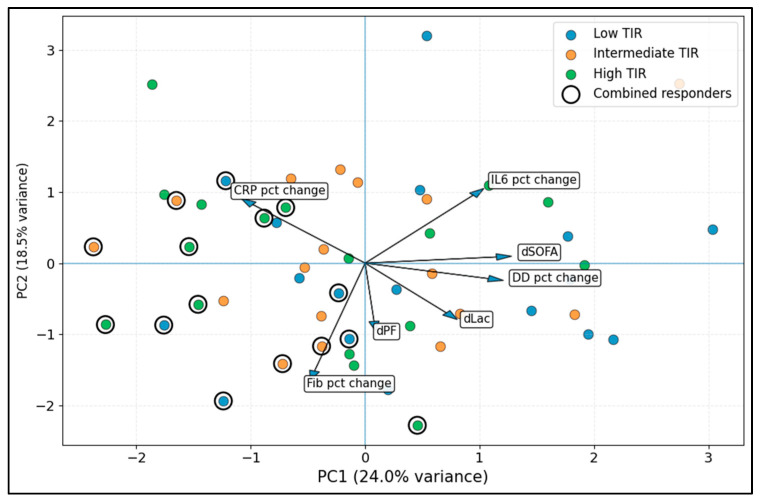
PCA map of multi-domain response patterns (biomarker + physiology change space). PC1, principal component 1; PC2, principal component 2; dSOFA, change in Sequential Organ Failure Assessment score; dLac, change in lactate; dPF, change in PaO_2_/FiO_2_; CRP pct change, C-reactive protein percent change; IL-6 pct change, interleukin-6 percent change; DD pct change, D-dimer percent change; Fib pct change, fibrinogen percent change; TIR, thrombo-inflammatory imbalance ratio.

The first two principal components (PC1 and PC2) captured 42.5% of total variance and provided a compact multidimensional response map. In this biplot, arrows indicate the direction of greater contribution from each change variable, and ringed points denote combined responders. Separation away from vectors representing worsening organ dysfunction or lactate accumulation and toward larger biomarker response vectors suggests a more favorable composite response pattern (Figure 4).

## 4. Discussion

### 4.1. Analysis of Findings

Our cohort underscores the heterogeneity of “COVID-19 sepsis” biology even within a relatively small, single-center TPE-treated population. The endothelial activation index (EAI = D-dimer/fibrinogen) separated two clinically similar severity strata (baseline SOFA/APACHE II and PaO_2_/FiO_2_ did not differ), yet with markedly different coagulation–inflammation profiles: the high-EAI phenotype had substantially higher D-dimer and lower fibrinogen, alongside unexpectedly lower CRP. This pattern is consistent with a more “endotheliopathy/immunothrombosis-forward” state where thrombin generation, fibrin turnover, and consumption dominate over a classic acute-phase signature. In severe COVID-19, cross-sectional evidence has linked critical illness to a constellation of endothelial injury/activation markers and prothrombotic imbalance (including heightened von Willebrand factor-related indices and platelet–endothelial activation), supporting the plausibility that a compact bedside surrogate such as EAI may partially reflect this biology [17]. In parallel, angiopoietin-2 (Ang-2)—a Tie2-pathway marker of endothelial activation—has been shown to predict ICU admission in hospitalized COVID-19, reinforcing that endothelial activation is not merely an epiphenomenon but a prognostically meaningful axis that may coexist with (or diverge from) CRP-dominant inflammation [18].

Using the ratio rather than D-dimer or fibrinogen alone allowed us to express fibrin turnover relative to substrate availability, which may better approximate endotheliopathy-dominant imbalance at the bedside while still preserving access to the individual component values.

Against that background, the phenotype-stratified peri-procedural changes suggest that TPE’s biochemical “footprint” may differ depending on whether the presenting syndrome is more thrombotic/endothelial vs. acute-phase inflammatory. High-EAI patients exhibited larger D-dimer reductions and a small but statistically significant median SOFA improvement (ΔSOFA −0.3 vs. +0.1), whereas low-EAI patients showed a far greater CRP decline despite less pronounced thrombotic-marker shifts. Mechanistic clinical observations support the notion that plasma exchange can reduce endotheliopathy-linked proteins and inflammatory mediators, yet the downstream physiologic translation may depend on whether the dominant pathogenic substrate is present and “exchangeable”. In a cohort of critically ill COVID-19 patients treated with plasma exchange, reductions in excess von Willebrand factor and inflammatory parameters were documented, aligning conceptually with an endotheliopathy-targeting rationale [19]. Similarly, retrospective ICU data have reported improvements in coagulation abnormalities (including fibrin degradation products) and suggested potential outcome signals—though with the usual limitations of non-randomized rescue therapy cohorts and the possibility of co-intervention/timing confounding [20].

At the same time, the observed SOFA signal should not be overinterpreted: although statistically significant, the absolute median change was small and is more appropriately viewed as a hypothesis-generating physiologic signal than a clinically decisive improvement [21,22].

A key signal in our analysis is that baseline IL-6 was the dominant predictor of achieving ≥50% IL-6 reduction: each doubling of baseline IL-6 increased the odds of response (OR ~1.94), while older age was associated with lower response probability. This pattern is biologically coherent and echoes broader COVID-19 literature showing that higher early cytokine levels (including IL-6) are independently associated with worse severity and survival, even after adjustment for clinical covariates, i.e., IL-6 behaves both as a marker of inflammatory burden and as a stratifier of a host-response state [23]. From a treatment-response standpoint, higher starting IL-6 may also create a “headroom effect” (both statistical and mechanistic) whereby large proportional declines are more attainable once the circulating cytokine burden is high. Importantly, the broader therapeutic landscape similarly supports the principle that matching therapy to an IL-6-driven phenotype matters: the WHO REACT prospective meta-analysis of randomized trials found an association between IL-6 antagonist use and lower mortality among hospitalized COVID-19 patients, emphasizing that targeting the IL-6 axis can improve outcomes when applied in appropriate contexts [24].

The observed coupling between IL-6 reduction and oxygenation improvement provides an additional layer of face validity for IL-6 change as more than a laboratory curiosity in at least a subset of patients. Across the cohort, IL-6 reduction (%) correlated positively with ΔPaO_2_/FiO_2_ and inversely with ΔCRP, suggesting that inflammatory de-escalation tracked with better gas exchange even when global organ failure metrics (ΔSOFA) were relatively static over the short observation window. Notably, interventional plasmapheresis data outside our setting also describe parallel improvements in inflammatory cytokines and oxygenation-related measures: in a controlled study of severe COVID-19, plasmapheresis reduced multiple cytokines (including IL-6) and was associated with improvements in clinical/laboratory parameters such as oxygen saturation and CRP, supporting a biologically plausible inflammation–oxygenation linkage [21]. Likewise, a clinical trial evaluating plasmapheresis in severe COVID-19 addressed cytokine-release modulation and survival-oriented endpoints, reinforcing that oxygenation and inflammatory trajectories are commonly co-assessed and may improve together even if the causal chain remains difficult to prove in complex ICU care [22].

One plausible mechanism linking IL-6 reduction to better oxygenation is attenuation of pulmonary vascular endothelial injury together with partial restoration of alveolar-capillary barrier function, which could reduce permeability edema and improve gas exchange before a broader change in global organ failure scores become evident [25,26].

Despite these biomarker and physiologic associations, hard outcomes in our cohort (ICU mortality, ventilator-free days, ICU length of stay) did not differ by IL-6 responder status or by combined phenotype–response strata, highlighting a familiar “biomarker–outcome disconnect” in rescue therapy observational datasets. This is not surprising given (i) small sample size, (ii) survivor/immortal-time and indication biases (patients must survive long enough to complete TPE and have paired labs), (iii) heterogeneity in timing, replacement fluids, and concomitant immunomodulators/anticoagulation, and (iv) the likelihood that once critical illness is entrenched, short-term biomarker shifts may be insufficient to reverse established lung and microvascular injury. The translational implication is not that phenotyping is futile, but that it likely must be more specific: coupling simple ratios (EAI) with direct endothelial markers (Ang-2, VWF/ADAMTS13 axis) that have demonstrated prognostic relevance [18,19]—and embedding this into prospective designs—may better identify the subgroup in whom extracorporeal “de-bulking” meaningfully intersects with the dominant pathobiology [17,20].

The lack of mortality separation between IL-6 responders and non-responders may reflect both limited statistical power and a true biomarker–outcome disconnect, whereby short-term cytokine improvement does not necessarily translate into survival benefit once multiorgan injury is established. Any potential biologic benefit of TPE must also be balanced against procedure-related risks, including catheter-related infection, bleeding, allergic reactions, electrolyte disturbances, and hemodynamic instability.

In critically ill COVID-19 patients receiving TPE as rescue therapy, a simple ratio-based phenotype (EAI = D-dimer/fibrinogen) may help clinicians interpret which biological axis is most “modifiable” during the peri-exchange window: low EAI aligned with pronounced acute-phase downshifts, whereas high EAI aligned with larger thrombotic-marker reduction and small SOFA improvement. Separately, baseline IL-6 appears most useful for anticipating a ≥50% IL-6 drop, suggesting that if IL-6 is being used as a treatment-response signal, “starting burden” matters and may guide expectations, monitoring cadence, and early reassessment—particularly when paired with oxygenation trends (IL-6 reduction correlated with ΔPaO_2_/FiO_2_).

Accordingly, EAI should be viewed as a pragmatic, hypothesis-generating enrichment tool rather than a standalone decision rule. Its most plausible clinical application is to support early identification of patients with a more endothelial/thrombotic profile in whom adjunctive TPE might merit closer consideration within standardized prospective protocols. Nevertheless, these findings should be interpreted in the appropriate clinical context, as specific patient factors, comorbidities, and cofounders may influence the results [27,28,29,30,31,32,33,34].

### 4.2. Study Limitations

This retrospective, single-center cohort lacked a contemporaneous non-TPE control group, precluding causal inference and making the observed peri-procedural changes vulnerable to confounding by the natural disease course and concomitant therapies. The sample size was modest (n = 51), limiting precision and power for hard clinical outcomes such as mortality and increasing the possibility of false-negative findings. TPE delivery was heterogeneous with respect to replacement fluid selection, exchange prescription adapted to body size and tolerance, and number of sessions, reflecting real-world rescue practice but adding treatment variability. Because inclusion required paired pre-/post-TPE measurements, patients who deteriorated early or died before repeat sampling may have been underrepresented, introducing potential survivor/selection bias. The retrospective dataset did not allow adjustment for important co-interventions such as anticoagulation intensity, corticosteroids, immunomodulators, or ventilatory strategy, and assay-specific measurement uncertainty was not available for modeling. Ethnicity was not consistently captured in the source records and therefore could not be analyzed reliably. Finally, EAI categories were derived from a cohort-specific median split and were not externally validated; thus, EAI should be regarded as an exploratory stratification tool pending confirmation in independent cohorts with prespecified thresholds and, ideally, a separate validation sample.

## 5. Conclusions

In this ICU cohort of severe COVID-19-associated sepsis treated with TPE, EAI (D-dimer/fibrinogen) distinguished two thrombo-inflammatory phenotypes with different peri-procedural biomarker trajectories, while baseline IL-6 was the strongest predictor of achieving a ≥50% IL-6 reduction, and older age reduced response odds. Although biomarker improvement, particularly IL-6 reduction, tracked with better oxygenation, clinical outcomes did not differ by responder status in this sample, supporting prospective, controlled studies that combine simple bedside ratios with validated endothelial markers and standardized timing to identify subgroups most likely to benefit.

Future studies should therefore use standardized TPE protocols, prospectively collected co-intervention data, and independent validation cohorts to determine whether phenotype-guided TPE can influence clinically meaningful outcomes.

## Figures and Tables

**Table 1:** Baseline characteristics by endothelial activation phenotype (EAI median split).

Variable	Low Thrombotic Activation (n = 25)	High Thrombotic Activation (n = 26)	*p*-Value
Age, years	49.0 (42.0–63.0)	53.5 (44.0–64.0)	0.617
Body mass index, kg/m^2^	29.0 (25.8–34.6)	27.9 (26.4–32.0)	0.799
Days from symptom onset to first TPE	10.5 (8.4–15.0)	11.1 (9.1–18.0)	0.327
APACHE II at baseline, points	8.5 (7.5–16.2)	11.5 (9.0–14.6)	0.327
SOFA at baseline, points	6.3 (4.4–10.0)	8.3 (4.3–12.0)	0.546
PaO_2_/FiO_2_ at baseline, mmHg	92.5 (66.5–169.4)	88.1 (61.9–162.0)	0.536
IL-6 at baseline, pg/mL	108.1 (7.6–599.0)	104.5 (19.5–425.6)	0.851
C-reactive protein at baseline, mg/L	159.0 (81.7–232.0)	31.8 (8.3–107.1)	<0.001
D-dimer at baseline, µg/mL	2.2 (1.1–4.7)	6.2 (3.7–7.9)	<0.001
Fibrinogen at baseline, g/L	7.1 (5.7–8.7)	2.9 (2.3–3.2)	<0.001
Lactate at baseline, mmol/L	2.3 (1.7–3.2)	2.3 (1.9–2.6)	0.604
Female sex	12/25 (48.0%)	4/26 (15.4%)	0.017
Diabetes mellitus	5/25 (20.0%)	3/26 (11.5%)	0.465
Arterial hypertension	13/25 (52.0%)	7/26 (26.9%)	0.089
Obesity	7/25 (28.0%)	9/26 (34.6%)	0.764
Chronic obstructive pulmonary disease	5/25 (20.0%)	2/26 (7.7%)	0.248

APACHE II, Acute Physiology and Chronic Health Evaluation II; IL-6, interleukin-6; PaO_2_/FiO_2_, arterial oxygen partial pressure to fraction of inspired oxygen ratio; SOFA, Sequential Organ Failure Assessment; TPE, therapeutic plasma exchange.

**Table 2:** Peri-procedural changes (Δ = post − baseline) by EAI phenotype.

Parameter	Low Thrombotic Activation (n = 25)	High Thrombotic Activation (n = 26)	*p*-Value
Δ IL-6, pg/mL	−15.8 (−132.0–−0.2)	−29.8 (−202.9–3.9)	0.917
IL-6 reduction, %	33.1 (7.2–53.3)	39.3 (−27.9–74.2)	0.992
Δ CRP, mg/L	−84.0 (−123.7–−23.0)	−2.3 (−53.6–14.9)	0.001
Δ D-dimer, µg/mL	−0.6 (−1.7–−0.1)	−2.5 (−4.7–−1.6)	0.004
Δ fibrinogen, g/L	−3.1 (−5.0–−2.3)	−0.4 (−0.6–0.4)	<0.001
Δ lactate, mmol/L	−0.2 (−0.6–0.4)	−0.3 (−0.7–0.1)	0.332
Δ PaO_2_/FiO_2_, mmHg	5.5 (−33.9–26.7)	8.0 (−13.1–25.3)	0.49
Δ MAP, mmHg	−2.0 (−7.9–2.1)	−3.1 (−5.0–−0.9)	0.992
Δ SOFA, points	0.1 (0.0–1.0)	−0.3 (−1.0–0.0)	0.026

Δ, change (post − baseline); CRP, C-reactive protein; IL-6, interleukin-6; MAP, mean arterial pressure; PaO_2_/FiO_2_, arterial oxygen partial pressure to fraction of inspired oxygen ratio; SOFA, Sequential Organ Failure Assessment.

**Table 3:** Baseline predictors and outcomes by IL-6 responder status (≥50% reduction).

Variable	IL-6 Responders (n = 20)	Non-Responders (n = 31)	*p*-Value
Age, years	52.3 (41.3–64.1)	51.0 (44.3–62.9)	0.692
Days from symptom onset to first TPE	13.0 (9.4–22.3)	10.4 (8.9–15.8)	0.15
SOFA at baseline, points	9.0 (5.3–12.0)	6.3 (3.0–10.3)	0.22
APACHE II at baseline, points	14.5 (8.8–15.2)	9.3 (8.3–13.7)	0.18
PaO_2_/FiO_2_ at baseline, mmHg	99.0 (68.5–170.0)	89.0 (52.9–147.3)	0.174
IL-6 at baseline, pg/mL	365.2 (107.6–832.3)	47.1 (8.0–184.6)	<0.001
C-reactive protein at baseline, mg/L	90.0 (63.9–205.2)	78.5 (8.6–163.9)	0.199
D-dimer at baseline, µg/mL	3.9 (2.4–7.5)	4.3 (2.1–6.5)	0.5
Endothelial activation index (D-dimer/fibrinogen)	1.0 (0.3–2.3)	0.8 (0.5–2.0)	0.931
Body mass index, kg/m^2^	27.9 (26.2–32.5)	29.0 (26.1–32.5)	0.664
Female	4/20 (20.0%)	12/31 (38.7%)	0.221
High thrombotic activation	11/20 (55.0%)	15/31 (48.4%)	0.776
ICU mortality	12/20 (60.0%)	16/31 (51.6%)	0.58
Ventilator-free days at day 28	0.0 (0.0–0.0)	0.0 (0.0–4.9)	0.084
ICU length of stay, days	33.6 (29.9–36.0)	31.7 (28.1–33.8)	0.186
Ventilation duration, days	28.0 (27.3–28.2)	27.1 (23.1–28.0)	0.088

APACHE II, Acute Physiology and Chronic Health Evaluation II; ICU, intensive care unit; IL-6, interleukin-6; PaO_2_/FiO_2_, arterial oxygen partial pressure to fraction of inspired oxygen ratio; SOFA, Sequential Organ Failure Assessment; TPE, therapeutic plasma exchange.

**Table 4:** Multivariable logistic regression for IL-6 responder status (≥50% IL-6 reduction).

Predictor	OR (95% CI)	*p*-Value
log_2_ baseline IL-6 (per doubling)	1.94 (1.27–2.95)	0.002
Endothelial activation index (per 1.0 unit)	1.16 (0.92–1.46)	0.212
Days from symptom onset to first TPE (per day)	1.18 (0.96–1.44)	0.114
Baseline SOFA (per 1 point)	0.86 (0.66–1.12)	0.255
Age (per year)	0.91 (0.84–0.99)	0.032
Baseline PaO_2_/FiO_2_ (per 10 mmHg)	1.12 (0.99–1.26)	0.073

CI, confidence interval; IL-6, interleukin-6; log_2_, base-2 logarithm; PaO_2_/FiO_2_, arterial oxygen partial pressure to fraction of inspired oxygen ratio; SOFA, Sequential Organ Failure Assessment; TPE, therapeutic plasma exchange.

**Table 5:** Spearman correlations between IL-6 reduction (%) and concurrent peri-procedural changes.

Pair (with IL-6 Reduction %)	Spearman ρ	*p*-Value
Δ CRP	−0.41	0.003
Δ D-dimer	−0.10	0.502
Δ SOFA	−0.03	0.825
Δ PaO_2_/FiO_2_	0.37	0.01
Δ MAP	−0.15	0.292
Δ lactate	0.12	0.383

Δ, change (post − baseline); CRP, C-reactive protein; IL-6, interleukin-6; MAP, mean arterial pressure; PaO_2_/FiO_2_, arterial oxygen partial pressure to fraction of inspired oxygen ratio; ρ, Spearman rank correlation coefficient; SOFA, Sequential Organ Failure Assessment.

**Table 6:** Outcomes by combined phenotype–response strata (EAI phenotype × IL-6 responder).

Outcome	Low EAI/IL-6 Responder	Low EAI/Non-Responder	High EAI/IL-6 Responder	High EAI/Non-Responder	*p*-Value (Overall)
Ventilator-free days at day 28	0.0 (0.0–0.0)	0.0 (0.0–4.9)	0.0 (0.0–0.0)	0.0 (0.0–3.3)	0.294
ICU length of stay, days	33.9 (29.9–35.8)	32.0 (27.8–34.0)	33.5 (30.0–35.2)	31.5 (28.2–33.7)	0.612
Ventilation duration, days	28.0 (27.9–28.0)	26.9 (23.1–28.0)	28.0 (26.9–28.5)	27.4 (22.8–28.0)	0.374
Δ SOFA, points (post–baseline)	1.0 (0.0–1.0)	0.1 (−0.2–1.0)	0.0 (−1.0–0.4)	−0.4 (−1.1–0.0)	0.142
IL-6 reduction, %	79.3 (53.3–97.8)	11.0 (−63.8–29.4)	80.0 (61.1–90.1)	−27.1 (−100.3–13.3)	<0.001
ICU mortality	6/9 (66.7%)	7/16 (43.8%)	6/11 (54.5%)	9/15 (60.0%)	0.69
SOFA improvement ≥ 1 point	2/9 (22.2%)	3/16 (18.8%)	5/11 (45.5%)	6/15 (40.0%)	0.382

Δ, change (post − baseline); ICU, intensive care unit; IL-6, interleukin-6; SOFA, Sequential Organ Failure Assessment.

## Data Availability

The data presented in this study are available on request from the corresponding author.
